# Genetic Determinants Highlight the Existence of Shared Etiopathogenetic Mechanisms Characterizing Age-Related Macular Degeneration and Neurodegenerative Disorders

**DOI:** 10.3389/fneur.2021.626066

**Published:** 2021-05-31

**Authors:** Claudia Strafella, Valerio Caputo, Andrea Termine, Carlo Fabrizio, Paola Ruffo, Saverio Potenza, Andrea Cusumano, Federico Ricci, Carlo Caltagirone, Emiliano Giardina, Raffaella Cascella

**Affiliations:** ^1^Genomic Medicine Laboratory UILDM, IRCCS Santa Lucia Foundation, Rome, Italy; ^2^Medical Genetics Laboratory, Department of Biomedicine and Prevention, Tor Vergata University, Rome, Italy; ^3^Department of Biomedicine and Prevention, Tor Vergata University, Rome, Italy; ^4^UOSD of Ophthalmology PTV Foundation “Policlinico Tor Vergata”, Rome, Italy; ^5^UNIT Retinal Diseases PTV Foundation “Policlinico Tor Vergata”, Rome, Italy; ^6^Department of Clinical and Behavioral Neurology, IRCCS Fondazione Santa Lucia, Rome, Italy; ^7^Department of Biomedical Sciences, Catholic University Our Lady of Good Counsel, Tirana, Albania

**Keywords:** age-related macular degeneration, neurodegenerative disorders, etiopathogenesis, susceptibility, miRNAs, genetic network

## Abstract

Age-related macular degeneration (AMD) showed several processes and risk factors in common with neurodegenerative disorders (NDDs). The present work explored the existence of genetic determinants associated with AMD, which may provide insightful clues concerning its relationship with NDDs and their possible application into the clinical practice. In this study, 400 AMD patients were subjected to the genotyping analysis of 120 genetic variants by OpenArray technology. As the reference group, 503 samples representative of the European general population were utilized. Statistical analysis revealed the association of 23 single-nucleotide polymorphisms (SNPs) with AMD risk. The analysis of epistatic effects revealed that *ARMS2, IL6, APOE*, and *IL2RA* could contribute to AMD and neurodegenerative processes by synergistic modulation of the expression of disease-relevant genes. In addition, the bioinformatic analysis of the associated miRNA variants highlighted miR-196a, miR-6796, miR-6499, miR-6810, miR-499, and miR-7854 as potential candidates for counteracting AMD and neurodegenerative processes. Finally, this work highlighted the existence of shared disease mechanisms (oxidative stress, immune-inflammatory response, mitochondrial dysfunction, axonal guidance pathway, and synaptogenesis) between AMD and NDDs and described the associated SNPs as candidate biomarkers for developing novel strategies for early diagnosis, monitoring, and treatment of such disorders in a progressive aging population.

## Introduction

One of the major consequences of the increasing life expectancy worldwide is the higher burden of age-related multifactorial diseases, including neurodegenerative disorders (NDDs) and cardiovascular, metabolic, and retinal disorders (1). In particular, NDDs and retinal disorders include a large spectrum of age-related conditions characterized by the progressive loss or dysfunction of neurons in specific areas of the central nervous system (CNS) and retina (2, 3). As a natural extension of the CNS, the retina shares several similarities with the brain and spinal cord concerning its anatomy, functionality, response to insult, and immunological features (2, 4). In addition, several that NDDs have manifestations in the eye as well as retinal diseases show different neurodegenerative processes similar to CNS-related conditions (2, 4, 5). These peculiarities led to refer to the eye as “a window to the brain,” since it can provide substantial information on brain health and disease and can enhance the research of new therapeutic avenues for the treatment of NDDs (2, 4). Indeed, both NDDs and age-related retinal disorders are characterized by a similar neurotoxic environment leading to oxidative and metabolic stress, excessive neuro-inflammatory response, mitochondrial dysfunction, and abnormal aggregation of proteins and debris, which, ultimately, cause the degeneration of neurons, photoreceptor cells, retinal ganglion cells, and their related axons (3, 4). Among the most investigated retinal disorders, age-related macular degeneration (AMD) showed several aging processes and risk factors in common with NDDs, including cognitive impairment, Alzheimer disease (AD), and Parkinson disease (PD) (2, 6). AMD is a multifactorial disease characterized by the progressive degeneration and the disruption of the cytoarchitectonics of the central portion of the retina. These damages can result from drusen formation and retinal pigment epithelium (RPE) changes in the early and intermediate stages of diseases. Later stages of AMD can be characterized by central geographic atrophy (non-exudative AMD) or abnormal choroidal neovascularization (CNV; known as exudative AMD) (6, 7). Concerning the etiopathogenetic background of AMD, a large number of studies identified several genetic and non-genetic variables affecting the susceptibility and the disease outcome in Italian and worldwide populations (8–12). Moreover, AMD patients were shown to be more susceptible to develop cognitive impairment, AD, and PD (13). Indeed, AMD, AD, and PD share different pathophysiological pathways, including protein misfolding and aggregation, microglia activation, oxidative stress, mitochondrial dysfunction, upregulated inflammatory response, complement activation, and imbalanced angiogenesis (2, 3, 13). In particular, despite that AMD is now being evaluated as an inflammatory condition where production of VEGF is a consequence of derailed innate and adaptive immunity, several evidences indicate an overactivation of microglia in AMD patients (14, 15). Microglia activation could be the result of the release of toxic substances deriving from drusen resorption reaching retina through breaches in external limiting membrane (15). Microglia overactivation would induce RPE alterations resulting in a more chemoattractive, pro-inflammatory, and pro-angiogenic environment that increases the recruitment and activation of immune cells and fosters the growth of neovascular vessels into the retina (16). Furthermore, the excess of inflammatory mediators accumulating within the retina would induce a toxic effect to neurons and macroglia, contributing to progressive retinal neurodegeneration (14, 17).

In this context, investigating the genetic background underlying AMD may shed light to the molecular components and pathways involved in the pathophysiology and susceptibility to NDDs and may be helpful for identifying biomarkers for monitoring early signs and symptoms of AD and PD in AMD patients. To this purpose, the present work aimed to explore the existence of genetic determinants associated with AMD, which may provide insightful clues concerning the relationship with NDDs and their possible application into the clinical practice. In particular, greater emphasis was given to non-coding variants, as they may modulate regulatory genetic and epigenetic networks relevant to both AMD and NDDs.

## Materials and Methods

### Study Subjects

The study cohort involved 400 Italian unrelated patients affected by exudative AMD, recruited from Retina Diseases UNIT at the PTV University Hospital of Rome. Patients were selected following specific inclusion criteria: male or female over the age of 50 years, clear media in order to guarantee good quality retina imaging, diagnosis of N-AMD supported by multimodal imaging evidences [fluorescein angiography, indocyanine angiography, and spectral domain–optical coherence tomography (OCT)]. Exclusion criteria were presence of any CNV type not related to AMD, high myopia, history of inherited retinal disease, allergy to fluorescein and/or indocyanine green, and unknown smoking habits. Recruited subjects were asked to reply to a questionnaire in order to classify their smoking habits. Subjects who have never smoked or quit smoking ≥5years before recruitment were classified as non-smokers; otherwise, they were considered as smokers. As the reference group, 503 samples representative of the European general population were derived from 1,000 Genomes and GnomAD databases.

None of the AMD patients had a diagnosis of NDDs at the moment of recruitment, as well as any information concerning reference subjects is available, since they have been retrieved by public databases. The research was approved by the ethical committee (CE/PROG.650 approved on 01/03/2018) of IRCCS Santa Lucia Foundation Hospital of Rome and was performed according to the Declaration of Helsinki. Written informed consent was obtained for all patients.

### Selection of Genetic Variants

Genetic variants have been selected considering the location within or nearby genes involved in cell proliferation, immune-inflammatory response, neuronal development, and angiogenesis as well as genes known to be involved in NDDs. To this purpose, literature data, Ensembl (GRCh38.p12 assembly), and PolymiRTs 3.0 database (18) were searched in order to extract 120 variants potentially involved in the regulation of gene expression and function. In particular, 91 rare and common variants were extracted from publicly available studies, including meta-analyses and genome-wide association studies (GWASs) focusing on NDDs and immune-inflammatory disorders. Keywords for searching literature studies were as follows: “neurodegenerative disease,” “Alzheimer's disease,” “Parkinson's disease,” “multiple sclerosis,” “neurodegeneration,” “neuroinflammation,” “genetic risk for neurodegenerative disorders,” and “immune response in neurodegenerative disorders.” Ensembl was utilized to check the frequency of variants, which were considered rare with a minor allele frequency (MAF) <0.1 and common with a MAF >0.1. In addition, 29 variants were selected from PolymiRTs 3.0 and Ensembl databases considering their location within genes coding for miRNAs and UTR of gene targets involved in NDDs. In particular, Polymirts 3.0 database allowed the sorting of 14 variants located within miRNA targeting genes associated with NDDs [namely, AD, PD, multiple sclerosis (MS), and amyotrophic lateral sclerosis (ALS)] from the list that is available from the database. Ensembl was also employed to assess the MAF of these variants in the European population and to select 15 single-nucleotide polymorphisms (SNPs) located within or nearby the genes associated with NDDs by literature (using the same keywords mentioned above). Moreover, the variants to be included into the panel have been also selected considering the availability of pre-designed TaqMan Assays on the manufacturer's site for each of the variants of interest, in order to avoid the design of custom TaqMan probes, which could not work on the OpenArray platform.

Finally, 12 coding variants and 108 variants in non-coding regions were selected for the present study. The list of variants selected for this study has been reported in Supplementary Table 1.

The existence of linkage disequilibrium (LD) patterns among variants located on the same chromosomes was evaluated in European samples derived from 1,000 Genomes database. LD analysis was performed through the LDmatrix tool of LDlink software (19), obtaining a heatmap matrix representing the LD patterns among the variants for each chromosome. Moreover, D′ and R2 values have been obtained for each pairwise LD as well. Considering the variants located in chromosome 1, high LD scores were obtained for rs2300747–rs1335532 (both within CD58 gene, D′ = 1.00, R2 = 0.90); rs1772159–rs823137 (mapping to *SLC41A1* and *RAB7L1*, respectively; D′ = 0.97, R2 = 0.87); and rs786843–rs1505067 (within *SEMA5A*, D′ = 1.00, R2 = 0.50). On chromosome 7, LD was detected for rs2280714 (*TNPO3*)–rs10954213 (*IRF5*), which reported a D′ = 1.00, R2 = 0.78. On chromosome 8, rs9331896–rs11136000 (both located within *CLU*) showed high LD (D′ = 0.97 and R2 = 0.91). Concerning chromosome 10, rs12722489–rs2075650 (within *IL2RA* gene) revealed total LD, with D′ = 1.000 and R2 = 0.589. On chromosome 19, the *APOE* variants rs429358–rs2075650 were found in partial LD, reporting a D′ = 0.760 and R2 = 0.480. On chromosome 20, rs2248359–rs2248137 (both located in *CYP24A1* gene) revealed high LD (D′ = 0.967 and R2 = 0.927). Finally, the *MAOA* variants rs1137070–rs2072743 located on chromosome X reported a high LD value, with D′ = 0.900 and R2 = 0.800.

### DNA Extraction and Quantification

Genomic DNA was extracted from 200–400 μl of whole blood with MagPurix Blood DNA Extraction Kit and MagPurix Automatic Extraction System (Resnova, Italy) according to the manufacturer's instructions. The concentration and quality of the extracted DNA were assessed by DeNovix Spectrophotometer (Resnova, Italy). In particular, DNA samples were characterized by a concentration range of 50–150 ng/μl, and A260/230 and A260/280 ratios included between 1.7 and 1.9.

### Genotyping Analysis

The genotyping analysis of the DNA samples was conducted by OpenArray Real-Time PCR technology on Quant Studio 12K Flex Real Time PCR System (Thermo Fisher Scientific, CA, USA). OpenArray technology utilizes TaqMan OpenArray plates with 3,072 through-holes, in which the TaqMan probes (Thermo Fisher Scientific, CA, USA) are spotted to obtain different formats. The customized panel of 120 assays specific for the selected variants enabled the simultaneous genotyping of 24 DNA samples per plate. For each sample, 30–150 ng of extracted DNA were re-suspended in 3 μl of pure distilled water and manually loaded into 384-well plates together with 3 μl of TaqMan OpenArray Genotyping Master Mix according to manufacturer's instructions. Negative controls were obtained by mixing water and Master Mix in a 1:1 ratio. The obtained mix was automatically transferred on the TaqMan OpenArray plates through the QuantStudio 12K Flex Accufill System. The loaded plates were then inserted into the QuantStudio 12K Flex Real Time PCR system (Thermo Fisher Scientific, CA, USA) to perform the Real-Time PCR run. Results were analyzed by the TaqMan Genotyper Software (Thermo Fisher Scientific, CA, USA) that enabled to perform the genotype calling and quality control. In particular, cluster normalization was performed with default parameters to normalize run-to-run variations in cluster positions caused by differences in reagent lots and experimental conditions. After normalization, the call rate (defined as the percentage of successful calls) was evaluated for each SNP considering a cutoff of 90%. Therefore, 15 SNP assays did not reach this threshold and thereby were excluded from further analyses. The removed variants were rs10074258, rs2672603, rs2234975, rs2075650, rs356219, rs786843, rs17174870, rs731236, rs1803274, rs6811520, rs1051643, rs45596840, rs10903832, rs20417, and rs76282929.

### Statistical Analysis

All of the statistical analyses were carried out using the R software (20). The variants derived from the quality control phase were tested for Hardy–Weinberg equilibrium (HWE) by means of two-sided Fisher's exact test at each locus. SNPs were considered in HWE with *p*-value > 0.05. Two-sided Fisher's exact tests were performed to compare allele frequencies between the two groups. The significance threshold was set at *p* < 0.05 and *q* < 0.05 to account for false discovery rate (21). Allelic odds ratios (ORs) with 95% confidence intervals were also estimated.

A state of art classification model was set up and validated with the purpose of discriminating between AMD and reference samples. An Extreme Gradient Boosting (XGBoost that combines many regression trees with a small learning rate) was used in combination with the DART algorithm, which is a dropout method from neural networks used to overcome the overspecialization issue in an Ensembl model of boosted regression trees (22). The train set consisted of 786 samples (322 AMD and 464 reference samples). Repeated k-fold cross-validation approach and area under the curve (AUC) metrics were used for model cross-validation and model evaluation. Finally, the model was tested on an independent holdout dataset of 87 samples (48 AMD and 39 reference samples). To assess the contribution of genetic and non-genetic factors to AMD susceptibility, associated SNPs were submitted to a multivariate logistic regression model, along with age, gender, and smoking habit. The train set for the model consisted of 659 samples (503 reference samples and 156 AMD). Repeated k-fold cross-validation was used to obtain 9/10 data folds with additional down-sampling strategy for model training and optimization in an inner cross-validation and to obtain 1/10 data folds for model testing in an outer cross-validation (23). The process was repeated five times, for each of the test data folds. Model performance was evaluated across resamples using AUC in receiver operating characteristic (ROC) curves. Moreover, the coefficient of determination (r2) was obtained using Zhang method based on the variance function (24). LD patterns among the associated variants were evaluated in the AMD cohort based on the location within the same chromosome.

To this purpose, LD and haplotype analyses were performed on Haploview 4.2 (https://www.broadinstitute.org/haploview/haploview) (25) with default parameters, and D′ and R2 scores were obtained for each pairwise LD. Results were visualized by generating a heatmap matrix. Genetic epistasis testing was performed among significant SNPs from Fisher's exact test using *W*-test, which can measure the association between binary phenotype and categorical genetic data (26). Only the main effects and pairwise variant interactions were tested. The obtained *p*-values were adjusted by means of Bonferroni correction set at *p* < 0.0005 and number of categorical combinations (k ≥ 8) (26).

### Bioinformatic Analyses

The significantly associated variants were tested for their potential effect on gene expression and function by means of bioinformatic tools. The variants located in the seed sequence of miRNAs were subjected to prediction analysis by ViennaRNAFold algorithm (27) and PolymiRTS (18) in order to evaluate the possible alteration of miRNA biogenesis or binding affinity with target mRNAs, respectively. The potential effect of miRNA variants on the stability of pre-miRNA secondary structures was evaluated by means of ViennaRNAfold algorithm that is available from ViennaRNA package 2.0. In particular, this tool allows predicting the secondary hairpin structures and computing its minimum free energy (MFE, ΔG). Wild-type and variant sequences of pre-miRNAs were retrieved from MiRBase (28) and tested by RNAfold tool in order to estimate differences in MFE that could affect the process of miRNAs biogenesis. Normally, the pre-miRNA structure with lower MFE is expected to be thermodynamically more stable and to enhance the processing into the mature miRNA. PolymiRTS predicted the impact of miRNA variants on miRNA–mRNA binding affinity through disruption or creation of binding sites. In addition, TargetScanHuman and miRPathDB were searched to identify the genes and pathways targeted by the associated miRNAs (29, 30). The set of associated genes was used as input for Gene Set Enrichment Analysis (GSEA) on g:GOSt tool of g:Profiler (31). The results of this analysis were clustered on the basis of their semantic similarity using default parameters on Revigo (32). The Ingenuity Pathway Analysis (IPA) and the generation of networks of interconnected genes and miRNAs were performed on IPA software (Qiagen, CA, USA). IPA is an all-in-one web-based software application that allows the analysis and integration of different kinds of genetic data, facilitating their interpretation, the identification of specific targets, or candidate biomarkers and placing them in the context of larger biological or chemical systems. The software is backed by the Ingenuity Knowledge Base, which consists of highly structured, detail-rich biological and chemical findings. In particular, Upstream Regulator Analysis, Disease and Functions, and Path Designer IPA tools were employed in this study. The Upstream Regulator Analysis was utilized to identify the genes, which may have a regulatory expression role and pinpoint network of genes interacting together, which could be implicated in specific biological pathways relevant to AMD and NDDs. The Disease and Functions tool was employed to categorize the genes associated with AMD into specific disease or pathophysiological mechanisms, which may explain the association between the gene and the disease. In this case, literature data were coupled with results retrieved from Disease and Functions IPA tool, in order to provide a more complete and accurate categorization based on predictive and experimental data. Finally, the Path Designer IPA tool was exploited to build and customize networks of interconnected genes and miRNAs and overlay them with canonical pathways, which may be related to the pathophysiology of AMD and NDDs.

## Results

### Statistical Association Analysis

**Figure 1:**
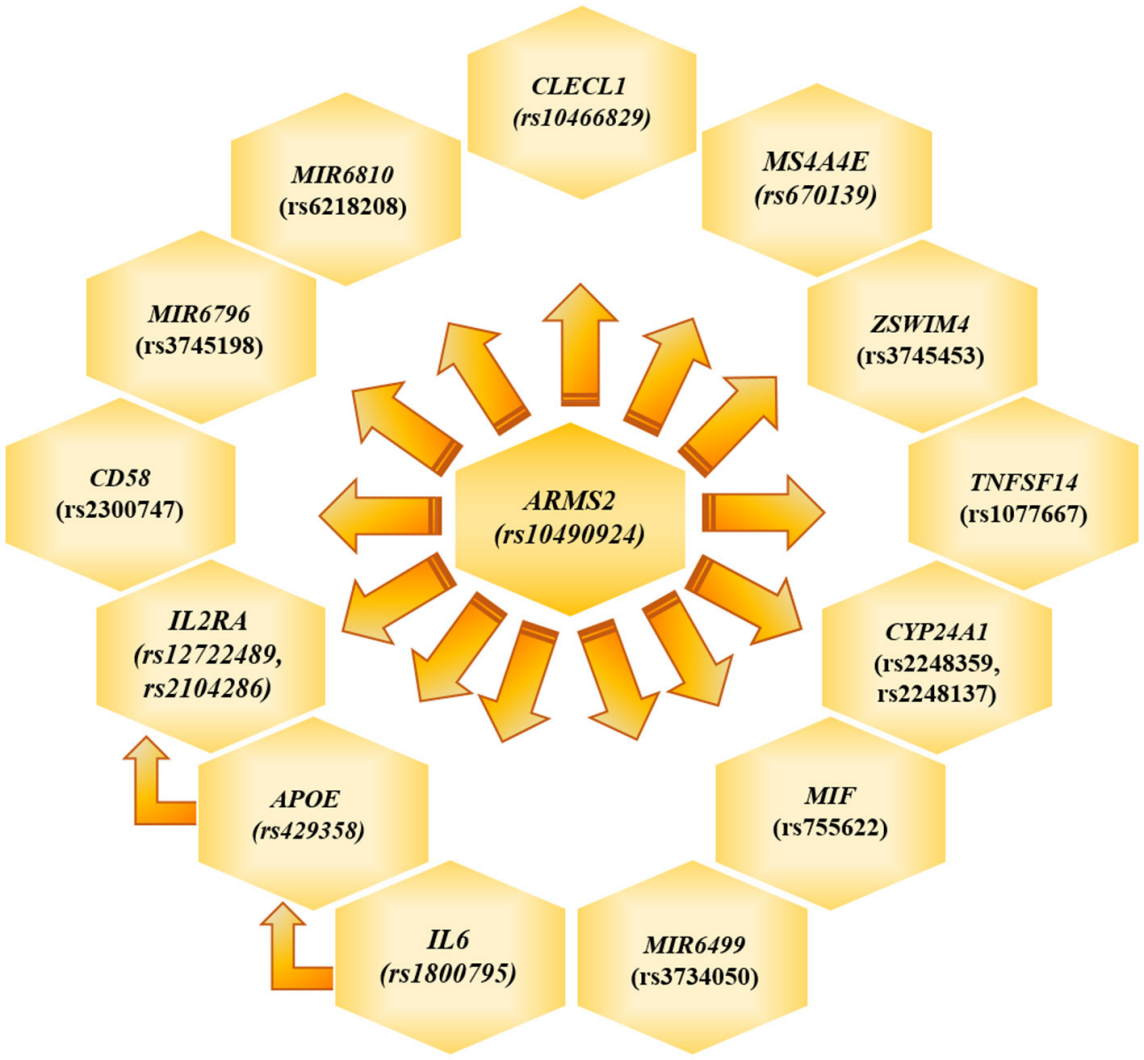
Genetic Epistasis. The figure illustrate the results referred to the genetic epistasis analysis, which highlighted *ARMS2* as key modulator gene able to affect the other surrounding genes and *APOE, IL6* and *IL2RA* as additional source of epistatic regulators.

The genotyping analysis reported 23 SNPs associated with AMD susceptibility in the studied cohort, as shown by the significant association values (*p* and *q* < 0.05) and ORs (Table 1 and Supplementary Table 2). In the present study, only significant data passing the *q* threshold were considered for further analyses. Successively, a classification model was generated by machine learning approaches, in order to assess the power of discrimination of the associated SNPs among AMD cases and reference samples. The classification model including the 23 associated variants reported an AUC value of 0.81, indicating that the associated variants are able to discriminate up to 81% of AMD cases with respect to reference subjects. Supporting this finding, the accuracy (0.77), sensitiveness (0.78), and specificity (0.75) of the model confirmed the good reliability and performance of the classification model. In addition, the multivariate logistic regression analysis (Table 2) identified six genetic variants (namely, rs10490924, rs1800795, rs429358, rs1077667, rs3745198, and rs755622) and the smoking habit as the most predictive factors for AMD risk in the present study, explaining 25% of the genetic variance in the studied cohort. LD patterns and genetic epistasis among the SNPs associated with AMD risk were evaluated. We evaluated the LD patterns for the SNPs located on the same chromosome in order to search for different LD patterns between cases and reference samples. As expected, the LD scores between rs2248359–rs2248137 (both located in *CYP24A1* gene) and rs12722489–rs2104286 (in *IL2RA* gene) in AMD samples (D′ = 0.942 and D′ = 0.975, respectively) were comparable with LD scores in reference subjects (D′ = 0.967 and D′ = 1.0, respectively). None of the other associated variants located on the same chromosome were in LD, meaning that they represent independent susceptibility markers for AMD. Given their independent association with the disease, we decided to evaluate the potential existence of genetic epistasis among the SNPs of interest. The analysis reported significant results for 17 SNPs out of 23 variants associated with AMD. The observed SNP–SNP interactions have been illustrated in Figure 1. In particular, the rs10490924 (*ARMS2*) presented the highest number of SNP–SNP interactions, suggesting its crucial role as a key epistatic modulator of a wider network of gene interactions. In addition, the epistasis analysis revealed significant SNP–SNP interactions among variants of *IL6, APOE*, and *IL2RA*, which may thereby provide additional information concerning the potential epistatic effects affecting the associated genes.

**Table 1:** SNPs significantly associated with AMD.

**SNP (gene)**	**Variant localization**	**Allele count in cases (frequency)**	**Allele count in ref subjects (frequency)**	***p*-value**	***q*-value**	**OR (95%CI)**
rs10490924, G/T (*ARMS2*)	Exonic	G: 462 (0.64) T: 262 (0.36)	G: 810 (0.80) T: 196 (0.20)	1.17 * 10^−14^	4.84 * 10^−13^	T = 2.34 (1.87–2.93)
rs1800795, C/G (*IL6*)	Intronic	C: 203 (0.28) G: 531 (0.72)	C: 418 (0.42) G: 588 (0.58)	2.09 * 10^−9^	5.78 * 10^−8^	G = 1.88 (1.51–2.32)
rs429358, T/C (*APOE*)	Exonic	T: 655 (0.93) C: 53 (0.07)	T: 850 (0.84) C: 156 (0.16)	4.16 * 10^−7^	8.61 * 10^−6^	T = 2.27 (1.63–3.22)
rs2248359, C/T (*CYP24A1*)	Regulatory region	C: 347 (0.48) T: 375 (0.52)	C: 597 (0.59) T: 409 (0.41)	4.03 * 10^−6^	6.68 * 10^−5^	T = 1.57 (1.29–1.92)
rs2248137, C/G (*CYP24A1*)	Intronic	C: 344 (0.49) G: 362 (0.51)	C: 599 (0.60) G: 407 (0.40)	1.10 * 10^−5^	0.0001	G = 1.54 (1.26–1.88)
rs2300747, A/G (*CD58*)	Intronic	A: 621 (0.92) G: 57 (0.08)	A: 863 (0.86) G: 143 (0.14)	0.0002	0.003	A = 1.81 (1.29–2.56)
rs11614913, C/T (*MIR196A2*)	Mature miRNA	C: 486 (0.68) T: 234 (0.32)	C: 593 (0.59) T: 413 (0.41)	0.0003	0.003	C = 1.44 (1.20–1.78)
rs2283792, T/G (*MAPK1*)	Intronic	T: 280 (0.40) G: 430 (0.60)	T: 483 (0.48) G: 523 (0.52)	0.0004	0.004	G = 1.42 (1.16–1.75)
rs12722489, C/T (*IL2RA*)	Intronic	C: 671 (0.91) T: 65 (0.09)	C: 863 (0.86) T: 143 (0.14)	0.0005	0.004	C = 1.72 (1.25–2.38)
rs1077667, C/T (*TNFSF14*)	Intronic	C: 602 (0.84) T: 116 (0.16)	C: 776 (0.77) T: 230 (0.23)	0.0006	0.004	C = 1.53 (1.20–2.0)
rs3745453, A/G (*ZSWIM4*)	3′UTR	A: 530 (0.75) G: 172 (0.25)	A: 683 (0.68) G: 323 (0.32)	0.0006	0.004	A = 1.47 (1.17–1.85)
rs3745198, C/G (*MIR6796*)	Mature miRNA	C: 419 (0.61) G: 263 (0.39)	C: 537 (0.53) G: 469 (0.47)	0.001	0.007	C = 1.41 (1.13–1.72)
rs35349669, C/T (*INPP5D*)	Intronic	C: 454 (0.62) T: 282 (0.38)	C: 543 (0.54) T: 463 (0.46)	0.001	0.008	C = 1.38 (1.13–1.69)
rs3734050, C/T (*MIR6499*)	Mature miRNA	C: 685 (0.93) T: 49 (0.07)	C: 897 (0.89) T: 109 (0.11)	0.003	0.01	C = 1.72 (1.20–2.50)
rs13401, G/A (*ATF6*)	3′UTR	G: 208 (0.28) A: 520 (0.71)	G: 225 (0.22) A: 781 (0.78)	0.003	0.01	G = 1.38 (1.10–1.73)
rs10889677, C/A (*IL23R*)	3′UTR variant	C: 457 (0.63) A: 263 (0.37)	C: 706 (0.70) A: 300 (0.30)	0.003	0.01	A = 1.35 (1.10–1.66)
rs2104286, T/C (*IL2RA*)	Intronic	T: 596 (0.84) C: 116 (0.16)	T: 785 (0.78) C: 221 (0.22)	0.003	0.01	T = 1.44 (1.12–1.88)
rs755622, G/C (*MIF*)	Intronic	G: 613 (0.87) C: 95 (0.13)	G: 819 (0.81) C: 187 (0.19)	0.004	0.01	G = 1.49 (1.12–1.96)
rs670139, G/T (*MS4A4E*)	Intronic	G: 461 (0.68) T: 221 (0.32)	G: 611 (0.60) T: 395 (0.40)	0.004	0.01	G = 1.35 (1.10–1.66)
rs10466829, G/A (*CLECL1*)	Intronic	G: 321 (0.44) A: 405 (0.56)	G: 515 (0.51) A: 491 (0.48)	0.004	0.01	A = 1.32 (1.10–1.61)
rs62182086, A/G (*MIR6810*)	Mature miRNA	A: 642 (0.90) G: 68 (0.10)	A: 866 (0.86) G: 140 (0.14)	0.006	0.02	A = 1.53 (1.12–2.12)
rs3746444, A/G (*MIR499A*)	Mature miRNA	A: 536 (0.75) G: 174 (0.25)	A: 811 (0.80) G: 195 (0.20)	0.01	0.04	G = 1.34 (1.10–1.71)
rs2925980, A/G (*MIR7854*)	Mature miRNA	A: 415 (0.62) G: 253 (0.38)	A: 685 (0.68) G: 321 (0.32)	0.01	0.04	G = 1.30 (1.10–1.60)

*OR, odds ratio; CI, confidence interval; Ref, reference; SNP, single-nucleotide polymorphism; AMD, age-related macular degeneration*.

**Table 2:** Multivariate regression analysis.

**SNP (gene)**	***p*-value**	**OR (95%CI)**
rs10490924, G/T (*ARMS2*)	0.03	T = 1.84 (1.10–3.22)
rs1800795, C/G (*IL6*)	0.04	G = 1.76 (1.00–3.11)
rs429358, T/C (*APOE*)	0.001	T = 3.05 (1.51–6.14)
rs1077667, C/T (*TNFSF14*)	0.01	C = 1.98 (1.12–3.50)
rs3745198, C/G (*MIR6796*)	0.009	C = 2.16 (1.21–3.88)
rs755622, G/C (*MIF*)	0.04	G = 1.82 (1.00–3.29)
Smoking	0.001	5.55 (1.96–16.0)

*OR, odds ratio; CI, confidence interval; SNP, single-nucleotide polymorphism*.

### Functional Analysis of Variants Located in MiRNA Genes

Six variants located in six miRNA genes were associated with AMD in the present study, namely, rs11614913 (*MIR196A2*), rs3745198 (*MIR6796*), rs3734050 (*MIR6499*), rs62182086 (*MIR6810*), rs3746444 (*MIR499A*), and rs2925980 (*MIR7854*). These variants were subjected to prediction analysis by ViennaRNAFold algorithm and PolymiRTS in order to evaluate the possible alteration of miRNA biogenesis or binding affinity with target mRNAs, respectively. In addition, TargetScanHuman and miRPathDB tools allowed finding the target genes of the miRNA associated with AMD in the present study. The prediction analysis by ViennaRNAFold reported that the secondary structures generated by the sequence carrying the risk alleles of rs11614913 (*MIR196A2*) and rs3745198 (*MIR6796*) had a lower MFE with respect to the structures generated by the alternative allele (Table 3). This finding indicates that the hairpin structure carrying the risk alleles may enhance the stability of the pre-miR-196a2 and pre-miR6796 and the subsequent processing and expression of the mature miR-196a and miR-6796. Concerning the other miRNA variants, the prediction of their impact on the secondary structures of the pre-miRNA sequence did not report significant differences. The analysis of the impact of miRNA variants on the binding affinity with target genes provided significant results for rs3745198 (miR-6796-3p), rs62182086 (miR-6810-5p), rs3746444 (miR-499a-3p), and rs2925980 (miR-7854-3p), for which the risk alleles were predicted to disrupt or create target sites for genes associated with AMD (Table 3). Finally, TargetScanHuman and miRPathDB tools allowed identifying different associated genes as targets of the miRNAs of interest and linking them with several biological pathways involved in the pathophysiology of AMD and NDDs (Table 3).

**Table 3:** Prediction analysis of the functional impact of miRNA variants associated with AMD.

**MiRNA**	**SNP (alleles)**	**Risk allele**	**MFE (allele)**	**Disrupted target sites by the variant allele**	**Created target sites by the variant allele**	**AMD-associated genes targeted by miRNAs**	**Biological pathways**
MiR-196a2-3p	rs11614913 (C/T)	C	MFE (C): −50.30 kcal/mol MFE (T): −44.70 kcal/mol	na	Na	*MAPK1, CD58, IL2RA, IL23R*	Neuroinflammation, neurodegeneration, oxidative stress, endoplasmic reticulum stress, synaptogenesis, endocytosis, unfolded protein response
MiR-196a2-5p			-	na	Na	*ATF6, MAPK1, TNFSF14*	
MiR-6796-3p	rs3745198 (C/G)	C	MFE (C): −32.30 kcal/mol MFE (G): −28.50 kcal/mol	-	*CYP24A1*, *MAPK1*	*ARMS2, ATF6, CD58, MAPK1, TNFSF14*	Cell motility, cell differentiation, signal transduction, neurogenesis, neuron differentiation, axon development, angiogenesis
MiR-6796-5p			-	-	-	*APOE, ATF6, CLECL1. INPP5D, MAPK1, TNFSF14, ZSWIM4*	
MiR-6499-3p	rs3734050 (C/T)	C	-	-	-	*ARMS2, ATF6, CD58, CYP24A1, IL2RA, IL23R, MAPK1, TNFSF14, ZSWIM4*	Mitochondrial function, vesicle trafficking, cell motility, signal transduction, regulation of cell death, neurogenesis, neuron differentiation, endoplasmic reticulum stress
MiR-6499-5p			MFE (C): −24.50 kcal/mol MFE (T): −25.20 kcal/mol	-	-	*-*	
MiR-6810-3p	rs62182086 (A/G)	A	-	-	-	*ATF6, MAPK1, TNFSF14, ZSWIM4*	Synaptogenesis signaling, neurogenesis, differentiation and projection of neurons, signal transduction
MiR-6810-5p			MFE (A): −30.20 kcal/mol MFE (G): −30.40 kcal/mol	*INPP5D*	*TNFSF14*	*APOE, ARMS2, ATF6, CD58, IL23R, INPP5D, MAPK1, TNFSF14*	
MiR-499a-3p	rs3746444 (A/G)	G	MFE (A): −63.20 kcal/mol MFE (G): −62.80 kcal/mol	*INPP5D*	-	*ATF6, CD58, MAPK1, INPP5D, TNFSF14*	Neuroinflammation, neurodegeneration, oxidative stress, endoplasmic reticulum stress, synaptogenesis, endocytosis, unfolded protein response
MiR-499a-5p			-	-	-	*ATF6, MAPK1*	
MiR-7854-3p	rs2925980 (A/G)	G	MFE (A): −26.40 kcal/mol MFE (G): −25.50 kcal/mol	-	*CD58, ZSWIM4, ATF6*	*ATF6, CD58, CYP24A1, MAPK1, TNFSF14, ZSWIM4*	Cell motility, cell differentiation, signal transduction, vesicle trafficking, synaptogenesis signaling
MiR-7854-5p			-	-	-	-	

*MFE, minimum free energy; AMD, age-related macular degeneration*.

### Gene Ontology Enrichment Analysis and Ingenuity Pathway Analysis

**Figure 2:**
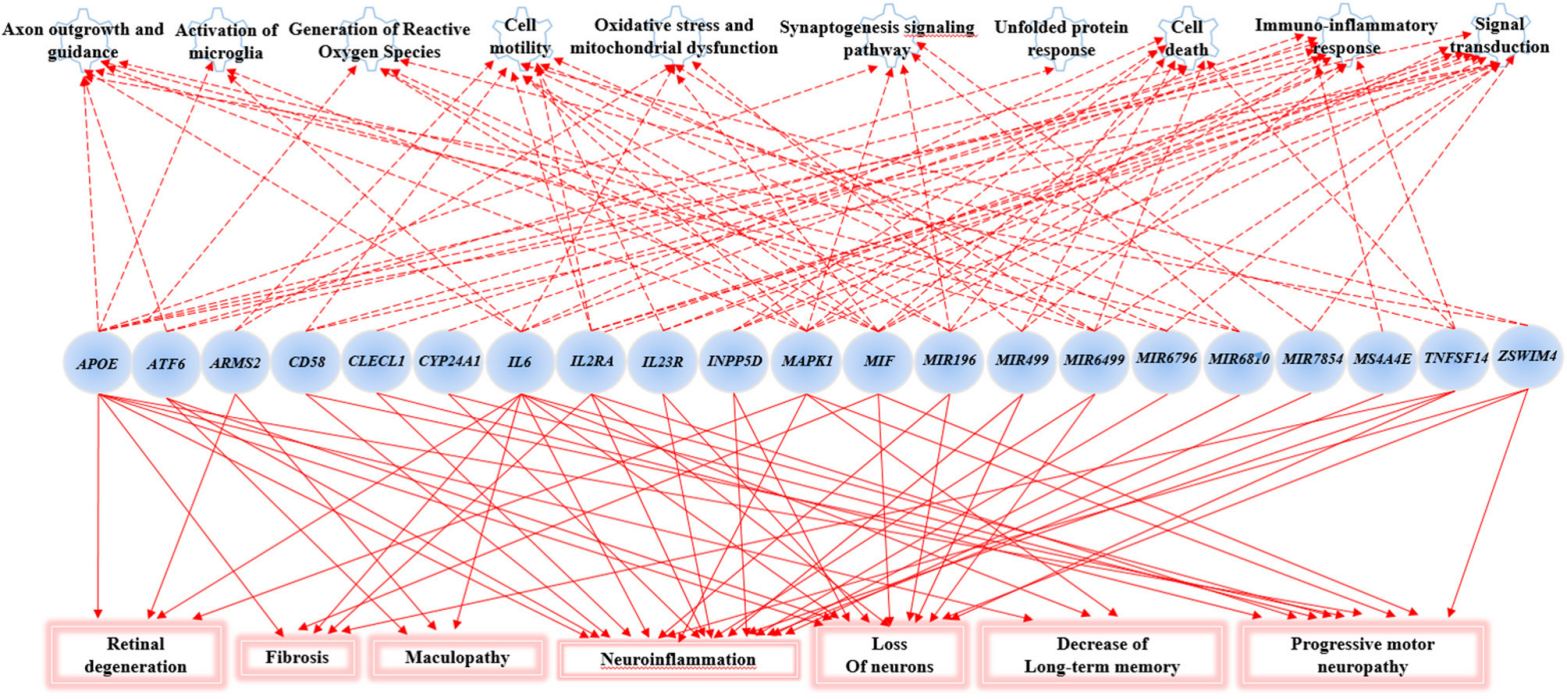
Gene Set enrichment analysis and Ingenuity Pathway Analysis (IPA). This figure shows the main biological pathways (upper side of the figure) and disease conditions (lower side of the figure) affected by the associated genes in the present study.

**Figure 3:**
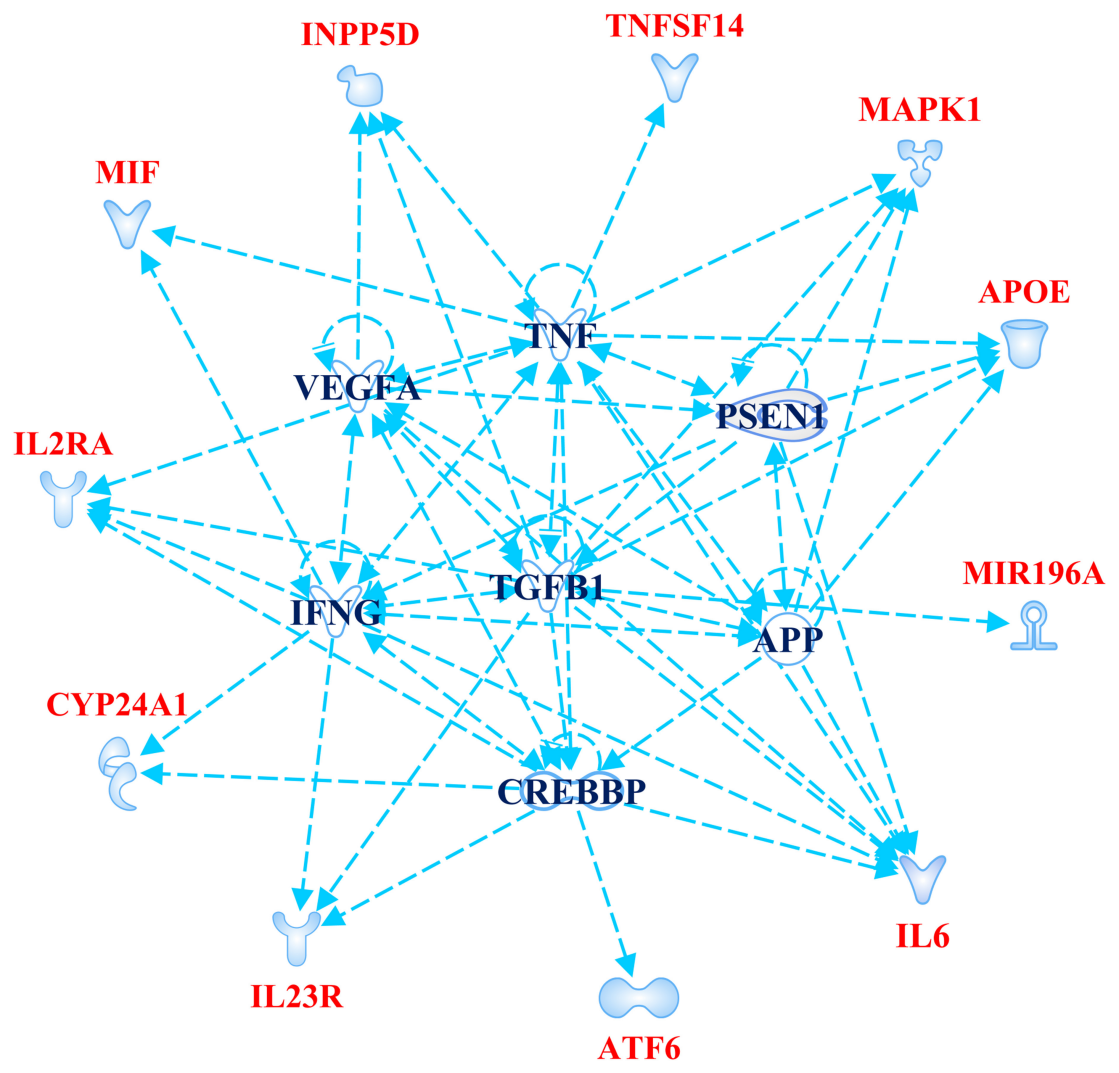
Upstream Regulator Analysis. The figure depicts the regulatory network of genes obtained by Upstream Regulator Analysis performed on IPA software (Qiagen). The network includes 11 age-related macular degeneration (AMD)-associated genes (highlighted in red color) and six upstream regulator molecules (highlighted in blue color). The figure has been created by Path Designer tool available on IPA software (Qiagen).

The Gene Ontology Enrichment (GOEA) and IPA (Qiagen) analyses allowed connecting the genes associated with AMD in the present study to different biological pathways underlying neuroinflammation and neurodegeneration (Figure 2). Interestingly, these pathways are common to the pathophysiology of AMD and NDDs. Supporting this finding, the “Disease And Functions” tool from IPA allowed categorizing the genes of interest into pathological conditions characterizing AMD (retinal degeneration, fibrosis, and maculopathy), NDDs (loss of neurons, decrease of long-term memory and progressive motor neuropathy), or both (neuroinflammation). Moreover, the Upstream Regulator Analysis by IPA tool revealed that the expression of 11 genes associated with AMD (*APOE, ATF6, CYP24A1, INNP5D, IL2RA, IL6, IL23R, MAPK1, MIF, MIRNA196A*, and *TNFSF14*) may be affected by a regulatory network including *APP, CREBBP, IFNG, PSEN1, TGFB1*, and *VEGFA* genes (Figure 3). In particular, among the affected mechanisms, the following are worth mentioning: neuroinflammation (mediated by HGMB1, p38 MAPK, HIF1α, and PI3/AKT signaling), synaptogenesis, apoptosis, sirtuin signaling, amyloid processing, angiogenesis (through VEGFA and IL-8 signaling), production of nitric oxide (NO) and reactive oxygen species (ROS), and axonal guidance pathways.

## Discussion

The present study explored the genetic component underlying AMD susceptibility with the purpose of identifying novel susceptibility biomarkers and highlighting the existence of common genetic features with the onset and progression of NDDs. The rationale of the study lies in the fact that the etiopathogenesis and progression of AMD and NDDs are characterized by similar neuroinflammatory and neurotoxic events. In particular, microglia overactivation has a key role in the initiation and protraction of the chronic neuroinflammation occurring in AMD and NDDs (14). In AMD, activated microglia have been found to accumulate in the subretinal space and have been associated with drusen-like accumulations, RPE degeneration, photoreceptor atrophy, and CNV (16). In NDDs, activated microglia have been implicated in the progressive neuronal loss and in synaptic pruning in response to protein aggregates (amyloid-β, hyperphosphorylated tau, and α-synuclein), dysfunction, or damage of neurons (33). Given these data, the identification of genetic risk factors for AMD and NDDs provide the potential for applying them for developing novel strategies for early diagnosis, monitoring, and treatment of these disorders in a progressive aging population. Among the 120 genetic variants subjected to genotyping, 23 SNPs resulted to be associated with AMD risk in the Italian population (Table 1). Interestingly, the classification model showed that these SNPs were able to discriminate up to 81% of AMD cases from reference subjects with a good reliability and performance rates. The multivariate logistic regression analysis (Table 2) identified rs10490924 (*ARMS2*), rs1800795 (*IL6*), rs429358 (*APOE*), rs1077667 (*TNFSF14*), rs3745198 (*MIR6796*), rs755622 (*MIF*), and smoking habit as the most predictive variables. As expected, smoking is a strong predictor of AMD risk, being one of the major environmental contributors to disease etiopathogenesis, because of the RPE damage and the reduction of choroidal blood flow caused by the release of oxidative compounds present in cigarettes (6, 34). Less clear, instead, is the association of smoking with NDDs, for which there exist controversial data (34).

Overall, the classification model and the multivariate logistic regression analysis highlighted that the associated SNPs could be utilized for creating a genetic risk model able to stratify at-risk individuals who may benefit early diagnosis and therapeutic treatments. On this subject, our previous work highlighted prominent differences between the Italian and global populations concerning the contribution of genetic and non-genetic risk factors to AMD susceptibility and progression (6). In fact, eight susceptibility genes (*CFH, ARMS2, IL-8, TIMP3, SLC16A8, VEGFA*, and *COL8A1*), old age, and smoking habit were shown to be risk factors for AMD in the Italian population (accounting for 29% of disease susceptibility), whereas female sex and regular consumption of fruits and vegetables appeared to be protective (9). Concerning the AMD risk in the worldwide populations, up to 34 loci have been estimated to explain 47–71% of genetic susceptibility, whereas non-genetic factors accounted for 19–37% of disease risk (6).

Furthermore, 21 SNPs of the 23 associated in the present study have never been investigated in AMD to our knowledge, representing thereby novel susceptibility factors. The rs10490924 (*ARMS2*) and rs429358 (*APOE*), instead, are well-known to be associated with AMD (9, 35, 36). *APOE* has been extensively investigated in several diseases, since its three main alleles (ε*4*, ε*3*, and ε*2*) carry differential risk or protection for several conditions, such as AD, PD, cardiovascular disease, AMD, obesity, chronic airway obstruction, type 2 diabetes, gallbladder disease, and liver disease (36).

*ARMS2* is considered a major contributor to the genetic susceptibility of AMD among Italian and worldwide populations (6, 8–10, 37). Intriguingly, the evaluation of epistatic effects among the SNPs associated in this study revealed that *ARMS2* could represent a key epistatic modulator of a network of genes (Figure 1), which may contribute to AMD and neurodegenerative processes by synergistic effects. Moreover, the evaluation of epistatic effects showed significant SNP–SNP interactions among variants of *IL6, APOE*, and *IL2RA*, representing thereby an additional source of modulation of the expression of disease-relevant genes. The evidence of epistatic interactions among the associated SNPs may also be useful for developing genetic risk models, which take into account not only variants with cumulative effect size on AMD susceptibility but also variants with synergistic epistatic effects.

Among the AMD-associated SNPs, six of them (rs11614913, *MIR196A2*; rs3745198, *MIR6796*; rs3734050, *MIR6499*; rs62182086, *MIR6810*; and rs3746444, *MIR499A*; rs2925980, *MIR7854*) were located in miRNA genes. As a matter of fact, structural or sequence variants in miRNA genes or in the 3′UTR of their target genes can alter the biogenesis or the binding affinity of miRNAs, affecting thereby the transcriptional profile of target genes and the miRNA–mRNA interactions (38–40). As a result, miRNA variants have been proposed as additional contributors to the onset and progression of complex disorders (39, 40). In a previous work, we showed that SNPs of *MIR27A* (rs11671784 and rs895819) and of *MIR146A* (rs2910164) were significantly associated with AMD risk in the Italian population (39). In particular, these SNPs have been predicted to affect the functional activity of their corresponding miRNAs, especially the interaction with specific targets and, thus, may contribute to the exacerbation of the angiogenic and inflammatory pathways underlying AMD physiopathology (39, 41). The present study extended the set of miRNAs variants associated with AMD in the Italian population, supporting the existence of a “genetics of the epigenetics” contributing to the onset and progression of disease (39). The novel miRNA variants have been predicted to alter the biogenesis of their related miRNAs and the binding affinity of different target genes associated with AMD and to affect several biological pathways relevant to AMD and neurodegeneration (Table 3). Given these results, miR-196a, miR-6796, miR-6499, miR-6810, miR-499, and miR-7854 represent potential candidates for counteracting AMD and neurodegenerative processes through the modulation of epigenetic elements implicated in the regulation of gene expression.

The novel susceptibility genes identified in this work support the existence of a population-specific impact of genomic variants on the disease onset and progression, which may also explain the variable prevalence of AMD across different countries. These results are consistent with the multifactorial etiology of AMD that is characterized not only by multiple contributing genes but also by the influence of environment and lifestyle conditions, which can change over time, affecting the natural selection of genetic variants and conferring differential susceptibility to multifactorial diseases (42–44). This is the reason why frequent genetic variants, which may have been selected in the past because of their neutral or low impact on protein function/expression, may have become risk alleles because of the modern environment and lifestyle changes (42, 43). This phenomenon can also be exemplified in the present study, where most of the associated SNPs displayed the highest frequency allele associated with the risk of AMD. Such peculiarity is typical of several multifactorial disorders over AMD, including AD, PD, MS, psoriasis, psoriatic arthritis, atopic eczema, and metabolic syndrome (42, 45–49). Consistent with the existence of a common genetic background affecting the susceptibility to AMD and NDDs, the susceptibility genes identified in this study have also been investigated in NDDs (mainly AD, PD, MS, and amyotrophic lateral sclerosis) and support the thesis that AMD patients are at higher risk of developing NDDs in their life. For this reason, it has been proposed that AMD could represent a preclinical sign for AD and PD, and identifying related-biomarkers could be useful to provide these patients with early diagnosis or treatments aimed to avoid or slow down harmful complications or permanent brain injuries (13). In this regard, the 23 SNPs identified in the present work could represent candidate biomarkers to this purpose. The genes related to these variants have been shown to participate in several biological pathways (Figure 2) essential for neurodevelopment and brain cognitive and motor functions, which are severely compromised in NDDs (50–56). In particular, a regulatory network of genes including 11 AMD-associated genes and six upstream regulator molecules (Figure 3) has been predicted to modulate several pathways involved in the development, differentiation, function, and response to stress of retina and neurons. Such regulatory network could be further investigated, in order to identify novel therapeutic options for the treatment of NDDs or the development of drug-repurposing strategies. Finally, the present work highlighted the existence of shared etiopathogenetic features between AMD and NDDs, which may be worth exploring in the perspective of the increasing life expectancy with profound medical implications and the need for an effective precision medicine approach for both disease conditions.

## Data Availability Statement

The original contributions presented in the study are included in the article/Supplementary Material, further inquiries can be directed to the corresponding author/s.

## Ethics Statement

The studies involving human participants were reviewed and approved by Ethics Committee of IRCCS Santa Lucia Foundation Hospital of Rome. The patients/participants provided their written informed consent to participate in this study.

## Author Contributions

CS, VC, and RC: conceptualization. VC and PR: methodology. CS, VC, AT, and CF: data analysis. FR: patient recruitment, imaging, and phenotyping. SP, AC, CC, EG, and RC: resources. CS, EG, and RC: supervision. CS: writing (original draft preparation). CS, VC, AC, FR, CC, EG, and RC: writing (review and editing). All authors have read and agreed to the published version of the manuscript.

## Conflict of Interest

The authors declare that the research was conducted in the absence of any commercial or financial relationships that could be construed as a potential conflict of interest.
