# Supplementary material for: Genetic Determinants Highlight the Existence of Shared Etiopathogenetic Mechanisms Characterizing Age-Related Macular Degeneration and Neurodegenerative Disorders
Source: Front Neurol. 2021 May 31;12:626066. doi: 10.3389/fneur.2021.626066 (PMC8200556; doi:10.3389/fneur.2021.626066)
Supplement: Supplementary file 1 [file Data_Sheet_1.PDF]

Supplementary Table 1. Selection of the panel of 120 genetic variants to be analyzed by Open Array technology. Variants have been selected considering their potential role in the regulation of gene expression and function by means of primary literature data research on Pubmed® and bioinformatic databases, namely Ensembl and PolymiRTs 3.0. Genomic location is referred to the GRCh38.p12 assembly of Ensembl database; bp: base pairs.

Variant ID	Gene	Reference/database
**rs12151791**	*AAK1*	Ensembl database
**rs4147929**	*ABCA7*	[1]
**rs76282929**	*ABCA7*	[2]
**rs12368653**	*AGAP2*	[3]
**rs11154801**	*AHI1*	[3]
**rs2075650**	*APOE*	[4]
**rs429358**	*APOE*	[5]
**rs63750066**	*APP*	[2]
**rs10490924**	*ARMS2*	[6]
**rs2672603**	*ARMS2*	Ensembl database
**rs3789327**	*ARNTL*	[7]
**rs13401**	*ATF6*	Ensembl database
**rs638405**	*BACE1*	[8]
**rs12212193**	*BACH2*	[3]
**rs2300603**	*BATF*	[3]
**rs1803274**	*BCHE*	[9]
**rs2028597**	*CBLB*	[3]
**rs3865444**	*CD33*	[10]
**rs1335532**	*CD58*	[3]
**rs2300747**	*CD58*	[11]
**rs9282641**	*CD86*	[3]
**rs7200786**	*CLEC16A*	[3]
**rs10466829**	*CLECL1*	[3]
**rs11136000**	*CLU*	[4]
**rs9331896**	*CLU*	[1]
**rs1046411**	*CNNM2*	Ensembl database
**rs4680**	*COMT*	[12-13]
**rs165599**	*COMT*	[12]
**rs6656401**	*CR1*	[1]
**rs2248137**	*CYP24A1*	[14]
**rs2248359**	*CYP24A1*	[3, 14]
**rs10903832**	*DIP2C*	Ensembl database
**rs2303759**	*DKKL1*	[3]
**rs2046748**	*DPP6*	[11]
**rs8093731**	*DSG2*	[10]
**rs10074258**	*EFNA5*	[15]
**rs11810217**	*EVI5*	[3]
**rs6964**	*GAK*	Ensembl database
**rs75548401**	*GBA*	[16]
**rs2243123**	*IL12A*	[3]
**rs2546890**	*IL12B*	[3]
**rs7517847**	*IL23R*	[11]
**rs10889677**	*IL23R*	[11]

**rs2104286**	*IL2RA*	[17]
**rs12722489**	*IL2RA*	[17]
**rs1800795**	*IL6*	[11]
**rs6897932**	*IL7R*	[3]
**rs2227306**	*IL8*	[6]
**rs35349669**	*INPP5D*	[10]
**rs2004640**	*IRF5*	[11]
**rs10954213**	*IRF5*	[11]
**rs1051643**	*LMNB1*	Ensembl database
**rs1491942**	*LRRK2*	[18]
**rs7238078**	*MALT1*	[3]
**rs228614**	*MANBA/ NFKB1*	[3]
**rs2072743**	*MAOA*	[12]
**rs1137070**	*MAOA*	[12]
**rs1799836**	*MAOB*	[13]
**rs2283792**	*MAPK1*	[3]
**rs2942168**	*MAPT*	[16]
**rs190982**	*MEF2C*	[10]
**rs17174870**	*MERTK*	[3]
**rs755622**	*MIF*	[19]
**rs3803808**	*MIR132*	PolymiRTs 3.0 database
**rs2632516**	*MIR142*	PolymiRTs 3.0 database
**rs2910164**	*MIR146A*	[20]
**rs221300**	*MIR153-2/PTPRN2*	PolymiRTs 3.0 database
**rs1893650**	*MIR155HG*	[21]
**rs72997425**	*MIR181C*	PolymiRTs 3.0 database
**rs718079**	*MIR196A1*	PolymiRTs 3.0 database
**rs11614913**	*MIR196A2*	[22]
**rs107822**	*MIR219A1*	PolymiRTs 3.0 database
**rs34772568**	*MIR29A*	PolymiRTs 3.0 database
**rs24168**	*MIR29A*	[23]
**rs2724377**	*MIR29C*	PolymiRTs 3.0 database
**rs2187473**	*MIR34C*	PolymiRTs 3.0 database
**rs11623267**	*MIR433, RTL1*	[24]
**rs45596840**	*MIR4482*	PolymiRTs 3.0 database
**rs3746444**	*MIR499A*	[22]
**rs3734050**	*MIR6499*	PolymiRTs 3.0 database
**rs3745198**	*MIR6796*	PolymiRTs 3.0 database
**rs62182086**	*MIR6810*	PolymiRTs 3.0 database
**rs2925980**	*MIR7854*	PolymiRTs 3.0 database
**rs1046994**	*MMRN1*	Ensembl database
**rs874628**	*MPV17L2*	[3]
**rs670139**	*MS4A4E*	[10]
**rs3803039**	*NAV3*	Ensembl database
**rs2070744**	*NOS3*	[25]
**rs1799983**	*NOS3*	[25]
**rs1520333**	*PKIA-AS/IL7*	[3]
**rs20417**	*PTGS2*	[26]

**rs28834970**	*PTK2B*	[10]
**rs4410871**	*PVT1/MYC*	[3]
**rs823137**	*RAB7L1*	Ensembl database
**rs180515**	*RPS6KB1*	[3]
**rs9722**	*S100B*	[27]
**rs1505067**	*SEMA5A*	Ensembl database
**rs786843**	*SEMA5A*	Ensembl database
**rs26595**	*SEMA6A*	[28]
**rs2234975**	*SIRT1*	Ensembl database
**rs10498633**	*SLC24A4/RIN3*	[10]
**rs1772159**	*SLC41A1*	Ensembl database
**rs356219**	*SNCA*	[16]
**rs11218343**	*SORL1*	[1]
**rs10201872**	*SP140*	[3]
**rs9891119**	*STAT3*	[3]
**rs729022**	*SYT11*	Ensembl database
**rs1738074**	*TAGAP*	[3]
**rs2293370**	*TIMMDC1/TMEM39A*	[3]
**rs6811520**	*TMEM165*	[7]
**rs1800693**	*TNFRSF1A*	[3]
**rs1077667**	*TNFSF14*	[3]
**rs2280714**	*TNPO3*	[11]
**rs4648356**	*TTC34/MMEL1*	[3]
**rs34725611**	*TYK2*	[3]
**rs731236**	*VDR*	[29]
**rs1044165**	*VSIG4*	[22, 30]
**rs6062314**	*ZBTB46*	[3]
**rs1250550**	*ZMIZ1*	[3]
**rs3745453**	*ZSWIM4*	[30]

REFERENCES

1. Nicolas, G., Charbonnier, C., & Campion, D. (2016). From Common to Rare Variants: The Genetic Component of Alzheimer Disease. *Human Heredity*, *81*(3), 129–141. https://doi.org/10.1159/000452256.
2. Jamal, S., Goyal, S., Shanker, A., & Grover, A. (2017). Computational Screening and Exploration of Disease-Associated Genes in Alzheimer's Disease. *Journal of Cellular Biochemistry*, *118*(6), 1471–1479. https://doi.org/10.1002/jcb.25806.
3. International Multiple Sclerosis Genetics Consortium, Wellcome Trust Case Control Consortium 2, Sawcer, S., Hellenthal, G., Pirinen, M., Spencer, C. C. A., Patsopoulos, N. A., Moutsianas, L., Dilthey, A., Su, Z., Freeman, C., Hunt, S. E., Edkins, S., Gray, E., Booth, D. R., Potter, S. C., Goris, A., Band, G., Oturai, A. B., … Compston, A. (2011). Genetic risk and a primary role for cell-mediated immune mechanisms in multiple sclerosis. *Nature*, *476*(7359), 214–219. https://doi.org/10.1038/nature10251.
4. Harold, D., Abraham, R., Hollingworth, P., Sims, R., Gerrish, A., Hamshere, M. L., Pahwa, J. S., Moskvina, V., Dowzell, K., Williams, A., Jones, N., Thomas, C., Stretton, A., Morgan, A. R., Lovestone, S., Powell, J., Proitsi, P., Lupton, M. K., Brayne, C., … Williams, J. (2009). Genome-wide association study identifies variants at CLU and PICALM associated with Alzheimer's disease. *Nature Genetics*, *41*(10), 1088–1093. https://doi.org/10.1038/ng.440.
5. Graetz, C., Gröger, A., Luessi, F., Salmen, A., Zöller, D., Schultz, J., Siller, N., Fleischer, V., Bellenberg, B., Berthele, A., Biberacher, V., Havla, J., Hecker, M., Hohlfeld, R., Infante-Duarte, C., Kirschke, J. S., Kümpfel, T., Linker, R., Paul, F., … Zipp, F. (2019). Association of smoking but not HLA-DRB1*15:01, APOE or body mass index with brain atrophy in early multiple sclerosis. *Multiple Sclerosis (Houndmills, Basingstoke, England)*, *25*(5), 661–668. https://doi.org/10.1177/1352458518763541.

6. Cascella R, Strafella C, Longo G, Ragazzo M, Manzo L, De Felici C, Errichiello V, Caputo V, Viola F, Eandi CM, Staurenghi G, Cusumano A, Mauriello S, Marsella LT, Ciccacci C, Borgiani P, Sangiuolo F, Novelli G, Ricci F, Giardina E (2017) Uncovering genetic and non-genetic biomarkers specific for exudative age-related macular degeneration: significant association of twelve variants. Oncotarget 9:7812-7821. https://doi.org/10.18632/oncotarget.23241.
7. Lavtar, P., Rudolf, G., Maver, A., Hodžić, A., Starčević Čizmarević, N., Živković, M., Šega Jazbec, S., Klemenc Ketiš, Z., Kapović, M., Dinčić, E., Raičević, R., Sepčić, J., Lovrečić, L., Stanković, A., Ristić, S., & Peterlin, B. (2018). Association of circadian rhythm genes ARNTL/BMAL1 and CLOCK with multiple sclerosis. *PloS One*, *13*(1), e0190601. https://doi.org/10.1371/journal.pone.0190601.
8. Wang ,M., Yang, J., Su, J. (2016). Relationship between the polymorphism in exon 5 of BACE1 gene and Alzheimer's disease. Aging Clin Exp Res, 29(2):105-113. doi: 10.1007/s40520-016-0539-0.
9. Reale, M., Costantini, E., Di Nicola, M., D'Angelo, C., Franchi, S., D'Aurora, M., Di Bari, M., Orlando, V., Galizia, S., Ruggieri, S., Stuppia, L., Gasperini, C., Tata, A. M., & Gatta, V. (2018). Butyrylcholinesterase and Acetylcholinesterase polymorphisms in Multiple Sclerosis patients: Implication in peripheral inflammation. *Scientific Reports*, *8*(1), 1319. https://doi.org/10.1038/s41598-018-19701-7.
10. Ridge, P. G., Hoyt, K. B., Boehme, K., Mukherjee, S., Crane, P. K., Haines, J. L., Mayeux, R., Farrer, L. A., Pericak-Vance, M. A., Schellenberg, G. D., Kauwe, J. S. K., & Alzheimer's Disease Genetics Consortium (ADGC). (2016). Assessment of the genetic variance of late-onset Alzheimer's disease. *Neurobiology of Aging*, *41*, 200.e13-200.e20. https://doi.org/10.1016/j.neurobiolaging.2016.02.024.
11. Tizaoui, K. (2018). Multiple sclerosis genetics: Results from meta-analyses of candidate-gene association studies. *Cytokine*, *106*, 154–164. https://doi.org/10.1016/j.cyto.2017.10.024
12. D'Amelio, M., Puglisi-Allegra, S., & Mercuri, N. (2018). The role of dopaminergic midbrain in Alzheimer's disease: Translating basic science into clinical practice. *Pharmacological Research*, *130*, 414–419. https://doi.org/10.1016/j.phrs.2018.01.016
13. Sampaio, T. F., Dos Santos, E. U. D., de Lima, G. D. C., Dos Anjos, R. S. G., da Silva, R. C., Asano, A. G. C., Asano, N. M. J., Crovella, S., & de Souza, P. R. E. (2018). MAO-B and COMT Genetic Variations Associated With Levodopa Treatment Response in Patients With Parkinson's Disease. *Journal of Clinical Pharmacology*, *58*(7), 920–926. https://doi.org/10.1002/jcph.1096.
14. Lu, M., Taylor, B. V., & Körner, H. (2018). Genomic Effects of the Vitamin D Receptor: Potentially the Link between Vitamin D, Immune Cells, and Multiple Sclerosis. *Frontiers in Immunology*, *9*, 477. https://doi.org/10.3389/fimmu.2018.00477.
15. Potkin, S. G., Guffanti, G., Lakatos, A., Turner, J. A., Kruggel, F., Fallon, J. H., Saykin, A. J., Orro, A., Lupoli, S., Salvi, E., Weiner, M., Macciardi, F., & Alzheimer's Disease Neuroimaging Initiative. (2009). Hippocampal atrophy as a quantitative trait in a genome-wide association study identifying novel susceptibility genes for Alzheimer's disease. *PloS One*, *4*(8), e6501. https://doi.org/10.1371/journal.pone.0006501.
16. Davis, A. A., Andruska, K. M., Benitez, B. A., Racette, B. A., Perlmutter, J. S., & Cruchaga, C. (2016). Variants in GBA, SNCA, and MAPT influence Parkinson disease risk, age at onset, and progression. *Neurobiology of Aging*, *37*, 209.e1-209.e7. https://doi.org/10.1016/j.neurobiolaging.2015.09.014.
17. Wang, X.-X., & Chen, T. (2018). Meta-analysis of the association of IL2RA polymorphisms rs2104286 and rs12722489 with multiple sclerosis risk. *Immunological Investigations*, *47*(5), 431–442. https://doi.org/10.1080/08820139.2018.1425699.
18. International Parkinson Disease Genomics Consortium, Nalls, M. A., Plagnol, V., Hernandez, D. G., Sharma, M., Sheerin, U.-M., Saad, M., Simón-Sánchez, J., Schulte, C., Lesage, S., Sveinbjörnsdóttir, S., Stefánsson, K., Martinez, M., Hardy, J., Heutink, P., Brice, A., Gasser, T., Singleton, A. B., & Wood, N. W. (2011). Imputation of sequence variants for identification of genetic risks for Parkinson's disease: A meta-analysis of genome-wide association studies. *Lancet (London, England)*, *377*(9766), 641–649. https://doi.org/10.1016/S0140-6736(10)62345-8.
19. Castañeda-Moreno, V. A., De la Cruz-Mosso, U., Torres-Carrillo, N., Macías-Islas, M. A., Padilla-De la Torre, O., Mireles-Ramírez, M. A., González-Pérez, O., Ruiz-Sandoval, J. L., Huerta, M., Trujillo, X., Ortuño-Sahagún, D., & Muñoz-Valle, J. F. (2018). MIF functional polymorphisms (-794 CATT5-8 and -173 G>C) are associated with MIF serum levels, severity and progression in male multiple sclerosis from western Mexican population. *Journal of Neuroimmunology*, *320*, 117–124. https://doi.org/10.1016/j.jneuroim.2018.04.006.
20. Zhou, Y., Chen, M., Simpson, S., Lucas, R. M., Charlesworth, J. C., Blackburn, N., van der Mei, I., Ponsonby, A.-L., Ausimmune/AUSLONG investigators group, & Taylor, B. V. (2018). Common genetic variation within miR-146a predicts disease onset and relapse in multiple sclerosis. *Neurological Sciences: Official Journal of the Italian Neurological Society and of the Italian Society of Clinical Neurophysiology*, *39*(2), 297–304. https://doi.org/10.1007/s10072-017-3177-1.
21. Paraboschi, E. M., Soldà, G., Gemmati, D., Orioli, E., Zeri, G., Benedetti, M. D., Salviati, A., Barizzone, N., Leone, M., Duga, S., & Asselta, R. (2011). Genetic association and altered gene expression of mir-155 in multiple sclerosis patients. *International Journal of Molecular Sciences*, *12*(12), 8695–8712. https://doi.org/10.3390/ijms12128695.

22. Kiselev, I., Bashinskaya, V., Kulakova, O., Baulina, N., Popova, E., Boyko, A., & Favorova, O. (2015). Variants of MicroRNA Genes: Gender-Specific Associations with Multiple Sclerosis Risk and Severity. *International Journal of Molecular Sciences*, *16*(8), 20067–20081. https://doi.org/10.3390/ijms160820067.
23. Li, Y., Li, C., Yang, M., Shi, L., Tao, W., Shen, K., Li, X., Wang, X., Yang, Y., Yao, Y. (2019). Association of single nucleotide polymorphisms of miRNAs involved in the GLUT4 pathway in T2DM in a Chinese population. Mol Genet Genomic Med, 7(9):e907. doi: 10.1002/mgg3.907.
24. Zhang, X., Yang, R., Hu, B.-L., Lu, P., Zhou, L.-L., He, Z.-Y., Wu, H.-M., & Zhu, J.-H. (2017). Reduced Circulating Levels of miR-433 and miR-133b Are Potential Biomarkers for Parkinson's Disease. *Frontiers in Cellular Neuroscience*, *11*, 170. https://doi.org/10.3389/fncel.2017.00170.
25. Heidari, M. M., Khatami, M., & Tahamtan, Y. (2017). Molecular Analysis of rs2070744 and rs1799983 Polymorphisms of NOS3 Gene in Iranian Patients With Multiple Sclerosis. *Basic and Clinical Neuroscience*, *8*(4), 279–284. https://doi.org/10.18869/nirp.bcn.8.4.279.
26. Chen, Q., Liang, B., Wang, Z., Cheng, X., Huang, Y., Liu, Y., & Huang, Z. (2016). Influence of four polymorphisms in ABCA1 and PTGS2 genes on risk of Alzheimer's disease: A meta-analysis. *Neurological Sciences: Official Journal of the Italian Neurological Society and of the Italian Society of Clinical Neurophysiology*, *37*(8), 1209–1220. https://doi.org/10.1007/s10072-016-2579-9
27. Fardell, C., Zettergren, A., Ran, C., Carmine Belin, A., Ekman, A., Sydow, O., Bäckman, L., Holmberg, B., Dizdar, N., Söderkvist, P., & Nissbrandt, H. (2018). S100B polymorphisms are associated with age of onset of Parkinson's disease. *BMC Medical Genetics*, *19*(1), 42. https://doi.org/10.1186/s12881-018-0547-3.
28. Xie, G., Roshandel, D., Sherva, R., Monach, P. A., Lu, E. Y., Kung, T., Carrington, K., Zhang, S. S., Pulit, S. L., Ripke, S., Carette, S., Dellaripa, P. F., Edberg, J. C., Hoffman, G. S., Khalidi, N., Langford, C. A., Mahr, A. D., St Clair, E. W., Seo, P., … Siminovitch, K. A. (2013). Association of granulomatosis with polyangiitis (Wegener's) with HLA-DPB1*04 and SEMA6A gene variants: Evidence from genome-wide analysis. *Arthritis and Rheumatism*, *65*(9), 2457–2468. https://doi.org/10.1002/art.38036.
29. Křenek, P., Benešová, Y., Bienertová-Vašků, J., & Vašků, A. (2018). The Impact of Five VDR Polymorphisms on Multiple Sclerosis Risk and Progression: A Case-Control and Genotype-Phenotype Study. *Journal of Molecular Neuroscience: MN*, *64*(4), 559–566. https://doi.org/10.1007/s12031-018-1034-1.
30. Ridolfi, E., Fenoglio, C., Cantoni, C., Calvi, A., De Riz, M., Pietroboni, A., Villa, C., Serpente, M., Bonsi, R., Vercellino, M., Cavalla, P., Galimberti, D., & Scarpini, E. (2013). Expression and Genetic Analysis of MicroRNAs Involved in Multiple Sclerosis. *International Journal of Molecular Sciences*, *14*(3), 4375–4384. https://doi.org/10.3390/ijms14034375.
