# Supplementary material for: Genetic Determinants Highlight the Existence of Shared Etiopathogenetic Mechanisms Characterizing Age-Related Macular Degeneration and Neurodegenerative Disorders
Source: Front Neurol. 2021 May 31;12:626066. doi: 10.3389/fneur.2021.626066 (PMC8200556; doi:10.3389/fneur.2021.626066)
Supplement: Supplementary file 2 [file Data_Sheet_2.PDF]

Supplementary Table 2: Genotype association analysis of the SNPs associated with AMD. Only Odd Ratios (OR) referred to the risk of disease have been reported. OR: Odd ratio, CI: Confidence Interval. Ref: Reference.

SNP (Gene)	Genotype count in cases (Frequency)	Genotype count in ref subjects (Frequency)	*p-value*	*q-value*	OR (95%CI)
**rs10490924, G/T (*ARMS2*)**	GG:160 (0.44)<br>GT:142 (0.39)<br>TT:60 (0.16)	GG:322 (0.64)<br>GT:166 (0.33)<br>TT:15 (0.03)	$1.93*10^{-14}$	$6.43*10^{-13}$	GT= 1.72 (1.26-2.33)<br>TT= 8.01 (4.33-15.70)
**rs1800795, C/G (*IL6*)**	CC:26 (0.07))<br>CG:151 (0.41)<br>GG:190 (0.52)	CC:96 (0.19)<br>CG:226 (0.45)<br>GG:181 (0.36)	$1.32*10^{-8}$	$2.94*10^{-7}$	CG= 1.58 (1.17-2.12)<br>GG= 4.00 (2.38-6.66)
**rs429358, T/C (*APOE*)**	TT:303 (0.86)<br>TC:49 (0.14)<br>CC:2 (0.005)	TT:358 (0.71)<br>TC:134 (0.26)<br>CC:11 (0.03)	$1.49*10^{-6}$	$1.99*10^{-6}$	TC= 2.32 (1.61-3.44)<br>TT= 4.76 (1.02-21.16)
**rs2248359, C/T (*CYP24A1*)**	CC:81 (0.23)<br>CT: 185 (0.51)<br>TT: 95 (0.26)	CC:173 (0.34)<br>CT:251 (0.50)<br>TT:79 (0.16)	$1.58*10^{-5}$	$1.50*10^{-4}$	CT= 1.57 (1.12-2.21)<br>TT= 2.56 (1.65-3.72)
**rs2248137, C/G (*CYP24A1*)**	CC:94 (0.26)<br>CG: 156 (0.45)<br>GG: 103 (0.29)	CC: 172 (0.34)<br>CG:255 (0.51)<br>GG:76 (0.15)	$3.85*10^{-6}$	$4.27*10^{-5}$	CG= 1.11 (0.80-1.56)<br>GG= 2.47 (1.26-1.88)
**rs2300747, A/G (*CD58*)**	AA:284 (0.84)<br>AG:53 (0.16)<br>GG:2 (0.006)	AA:372 (0.74)<br>AG:119 (0.24)<br>GG:12 (0.02)	0.001	0.006	AG= 1.72 (1.19-2.56)<br>AA= 4.76 (1.01-20.63)
**rs11614913, C/T (*MIR196A2*)**	CC:162 (0.45)<br>CT:162 (0.45)<br>TT:36 (0.10)	CC:182 (0.36)<br>CT:229 (0.45)<br>TT:92 (0.18)	$8.71*10^{-4}$	0.005	CT= 1.26 (0.93-1.72)<br>CC= 2.27 (1.44-3.70)
**rs2283792, T/G (*MAPK1*)**	TT:57 (0.16)<br>TG:166 (0.47)<br>GG:132 (0.37)	TT:127 (0.25)<br>TG:229 (0.46)<br>GG:147 (0.29)	0.001	0.009	TG= 1.25 (0.90-1.72)<br>GG= 2.00 (1.33-3.03)
**rs12722489, C/T (*IL2RA*)**	CC:304 (0.83)<br>CT:63 (0.17)<br>TT:1 (0.002)	CC: 374 (0.74)<br>CT:115 (0.23)<br>TT:14 (0.03)	$8.58*10^{-4}$	0.005	CT= 1.49 (1.04-2.12)<br>CC= 12.5 (1.72-50.00)
**rs1077667, C/T (*TNFSF14*)**	CC:249 (0.69)<br>CT:104 (0.29)<br>TT:6 (0.02)	CC:303 (0.60)<br>CT:170 (0.34)<br>TT:30 (0.06)	$8.35*10^{-4}$	0.005	CT= 1.35 (1.00-1.85)<br>CC= 4.16 (1.66-12.5)
**rs3745453, A/G (*ZSWIM4*)**	AA:205 (0.59)<br>AG:120 (0.34)<br>GG:26 (0.07)	AA:229 (0.45)<br>AG:225 (0.44)<br>GG:49 (0.01)	0.001	0.006	AG= 1.66 (1.25-2.27)<br>AA= 1.69 (1.00-2.94)
**rs3745198, C/G (*MIR6796*)**	CC:126 (0.37)<br>CG:167 (0.49)<br>GG:48 (0.14)	CC:142 (0.28)<br>CG:253 (0.50)<br>GG:108 (0.22)	0.003	0.01	CG= 1.35 (1.00-1.88)<br>CC= 2.00 (1.29-3.12)
**rs35349669, C/T (*INPP5D*)**	CC:148 (0.40)<br>CT:158 (0.43)<br>TT:62 (0.17)	CC:149 (0.30)<br>CT:245 (0.48)<br>TT:109 (0.22)	0.004	0.01	CT= 1.56 (1.13-2.12)<br>CC= 1.75 (1.17-2.63)
**rs3734050, C/T (*MIR6499*)**	CC:318 (0.87)<br>CT:49 (0.13)<br>TT:0 (0.00)	CC:403 (0.80)<br>CT:91 (0.18)<br>TT:9 (0.01)	0.002	0.01	CT= 1.47 (1.00-2.22)<br>CC= ∞
**rs13401, G/A (*ATF6*)**	GG:31 (0.09)<br>GA:146 (0.40)<br>AA: 187 (0.51)	GG:31 (0.06)<br>GA:163 (0.32)<br>AA:309 (0.62)	0.01	0.03	GA= 1.47 (1.09-1.99)<br>GG= 1.65 (1.00-2.90)
**rs10889677, C/A (*IL23R*)**	CC:138 (0.38)<br>CA:181 (0.50)<br>AA:41 (0.12)	CC:246 (0.50)<br>CA:214 (0.42)<br>AA:43 (0.08)	0.007	0.02	CA= 1.50 (1.11-2.03)<br>AA= 1.69 (1.02-2.81)
**rs2104286, T/C (*IL2RA*)**	TT:240 (0.83)<br>TC:116 (0.16)<br>CC: 0 (0.00)	TT:314 (0.73)<br>TC:157 (0.25)<br>CC:32 (0.01)	$1.46*10^{-7}$	$2.43*10^{-6}$	TC= 1.10 (0.70-1.40)<br>TT= ∞
**rs755622, G/C (*MIF*)**	GG:264 (0.75)<br>GC:85 (0.24)<br>CC:5 (0.01)	GG:332 (0.66)<br>GC:155 (0.31)<br>CC:16 (0.03)	0.01	0.04	GC= 1.47 (1.05-2.04)<br>GG= 2.56 (1.00-9.09)

**rs670139, G/T (*MS4A4E*)**	GG:159 (0.46)<br>GT:143 (0.42)<br>TT: 39 (0.11)	GG:193 (0.38)<br>GT:225 (0.45)<br>TT:85 (0.17)	0.02	0.04	GT= 1.29 (1.00-1.76)<br>GG= 1.81 (1.14-2.85)
**rs10466829, G/A (*CLECL1*)**	GG:69 (0.19)<br>GA:181 (0.50)<br>AA:111 (0.31)	GG:132 (0.26)<br>GA:251 (0.50)<br>AA:120 (0.24)	0.01	0.04	GA= 1.39 (1.00-2.00)<br>AA= 1.76 (1.17-2.66)
**rs62182086, A/G (*MIR6810*)**	AA:292 (0.83)<br>AG:58 (0.16)<br>GG:5 (0.01)	AA:369 (0.73)<br>AG:128 (0.25)<br>GG:6 (0.01)	0.004	0.01	AG= 1.75 (1.17-2.56)<br>AA= 1.10 (0.25-4.18)
**rs3746444, A/G (*MIR499A)***	AA:200 (0.56)<br>AG:136 (0.38)<br>GG:19 (0.06)	AA:330 (0.65)<br>AG:151 (0.42)<br>GG:22 (0.04)	0.02	0.05	AG= 1.48 (1.10-2.00)<br>GG= 1.42 (0.70-2.83)
**rs2925980, A/G (*MIR7854)***	AA:135 (0.40)<br>AG:145 (0.43)<br>GG:54 (0.16)	AA:225 (0.44)<br>AG:235 (0.47)<br>GG:43 (0.08)	0.003	0.01	AG=1.02 (0.75-1.40)<br>GG= 2.08 (1.29-3.38)
